# The usual and preferred sources of healthcare for Aboriginal and Torres Strait Islander Australians: a multilevel analysis

**DOI:** 10.1186/s12913-026-14526-x

**Published:** 2026-04-14

**Authors:** Feleke Hailemichael Astawesegn, Subash Thapa, Setognal B. Aychiluhm, Phil Naden, Zekariyas S. Nezenega, Cheru T. Leshargie, Zemenu Yohannes Kassa, Kedir Y. Ahmed

**Affiliations:** 1 Rural Health Research Institute, Charles Sturt University, Orange, NSW Australia; 2 College of Medicine and Health Sciences, Hawassa University, Hawassa, Ethiopia; 3 Institute of Public Health, College of Medicine and Health Sciences, University of Gondar, Gondar, Ethiopia; 4 Coonamble Aboriginal Health Service, Coonamble, NSW Australia; 5 Department of Health System Management and Health Policy, School of Public Health, Addis Ababa University, Addis Ababa, Ethiopia; 6 Discipline of General Practice, School of Medicine, Adelaide University, Adelaide, SA, Australia; 7 Translational Health Research Institute, Western Sydney University, Sydney, NSW Australia

**Keywords:** Aboriginal and Torres Strait Islander peoples, Sources of healthcare, Australia

## Abstract

**Background:**

Improving the health and well-being of Aboriginal and Torres Strait Islander Australians (hereafter referred to as Indigenous Australians) remains a national priority. While many mainstream services are already in place, Aboriginal Community Controlled Health Services (ACCHSs, hereafter referred to as Aboriginal Medical Services [AMSs]) are primarily established to provide Indigenous Australians with culturally safe, trusted, affordable, and holistic care that mainstream services often cannot offer. This study examines whether Aboriginal Medical Services/Community Centres (AMS/CC) are the usual and preferred sources of healthcare for Indigenous Australians and identifies the factors influencing these choices.

**Methods:**

This study used data from the 2018–19 National Aboriginal and Torres Strait Islander Health Survey (NATSIHS). We analysed responses from 4,416 Indigenous adults (usual source of care) and 4,712 adults (preferred source of care). Multilevel mixed-effects logistic regression was used to examine the association between individual and contextual factors with the choice of Aboriginal Medical Services/Community Controlled Health Services (AMS/ACCHS) as the usual and preferred sources of healthcare.

**Results:**

Our findings showed that most Indigenous Australians usually visited a mainstream GP (53%) more often than an AMS/CC (34%), while a higher proportion preferred AMS/CC (47%) over mainstream GPs (43%). The use of and preference for AMS/CC were higher among those experiencing financial stress (use: aOR = 2.25, 95% CI: 1.71–2.96; preference: aOR = 1.60, 95% CI 1.30–1.97), those satisfied with their access to cultural knowledge (use: aOR = 1.90, 95% CI: 1.38–2.61; preference: aOR = 1.40, 95% CI: 1.11–1.76), and individuals with a history of being removed from their natural family (use: aOR = 1.83, 95% CI: 1.33–2.52; preference: aOR = 1.70, 95% CI: 1.32–2.18). Experiencing discrimination in the past 12 months also increased the likelihood of using (aOR = 1.60, 95% CI: 1.20–2.13) or preferring (aOR = 1.68, 95% CI: 1.35–2.08) AMS/CC. Furthermore, Indigenous Australians living in inner or outer regional areas, very remote areas, and the Northern Territory were more likely to use or prefer AMS/CC. Conversely, those residing in less disadvantaged areas were less likely to use AMS/CC services than those in the most disadvantaged areas.

**Conclusions:**

Since most Indigenous Australians still use mainstream GPs, this probably reflects issues with availability and access, as AMSs are not accessible everywhere, leaving people with little option other than to see a GP. The findings highlight the need to expand AMSs, especially in rural and regional areas, and to tailor mainstream healthcare to ensure services are culturally appropriate, respectful, and aligned with the values and preferences of Indigenous communities.

## Background

Improving the health and well-being of Indigenous Australians remains a national priority [1, 2]. Despite recent progress, significant health inequities persist. Between 2020 and 2022, life expectancy at birth was 71.9 years for male Indigenous Australians compared to 80.6 years for male non-Indigenous Australians, while for females it was 75.6 years for Indigenous Australians versus 83.8 years for non-Indigenous Australians [3]. In 2018, the overall burden of disease for Indigenous Australians was reported as 2.3 times that of non-Indigenous Australians [4]. Key contributors included mental and substance use disorders, injuries, and chronic conditions such as cardiovascular disease, cancer, musculoskeletal disorders, and respiratory illnesses [4]. Addressing these challenges requires sustained, culturally responsive, and community-led approaches that build on Indigenous strengths, knowledge systems, and resilience [5].

The healthcare system plays a key role in supporting population health by providing appropriate, high-quality and timely care [6]. Australia’s healthcare system, which combines public and private sectors in both funding and service provision, is widely regarded as comprehensive and effective [7]. Nevertheless, barriers remain for many Indigenous Australians. For example, 3 in 10 (30%) Indigenous Australians who needed to visit a healthcare provider did not do so [4, 8, 9]. Reported barriers include institutional racism, geographic isolation, limited service availability, transport and distance challenges, shortage of Indigenous health professionals [10], socioeconomic disadvantage, and experiences of culturally inappropriate care [9, 11–17].

To address these challenges, the Australian Government has supported Indigenous-specific primary healthcare services, including ACCHS and community clinics serving populations with the greatest health and social needs [18]. ACCHS, established in 1971 with the first service in Redfern, are distinctive in being initiated, planned, and governed by Indigenous communities, ensuring culturally safe and holistic care [19, 20]. By 2022–23, 213 Indigenous-specific primary health care organisations were operating nationwide [19], funded primarily through the Indigenous Australians’ Health Programme, which allocated about 83% of its $4 billion budget between 2020 and 21 and 2023–24 to these services [21–23, 24].

While previous studies have highlighted the value of ACCHS and Community Clinics compared with mainstream health services [23, 25, 26] less is known about two key issues: [1] the healthcare settings Indigenous Australians usually access and prefer, and [2] the individual and community factors that influence their choice between ACCHS/Community Clinics and mainstream GPs or hospitals. Therefore, this study aimed to explore both the usual and preferred sources of healthcare, as well as the factors shaping these choices. Understanding these factors is essential for policymakers, healthcare providers, and Indigenous health organisations to strengthen Indigenous-led healthcare services and advance efforts to close the First Nations Health Gap [27, 28].

## Methods

### Data source and study design

We used data from the 2018-19 National Aboriginal and Torres Strait Islander Health Survey (NATSIHS) to examine Indigenous Australians’ sources of healthcare [29]. NATSIHS is a population-based survey conducted in collaboration with Australian Government agencies, state/territory government agencies, non-government organisations, and academic and research institutions [29]. The survey collects a wide range of information about the health and well-being of Indigenous Australians, including demographics, nutrition, social determinants, chronic diseases, experiences of self-harm and sources of healthcare care [30]. Data were collected via face-to-face interviews with those living in private dwellings across Australia. Detailed information about the survey methods is available elsewhere [29].

### Study population

The NATSIHS was conducted between July 2018 and April 2019. Two samples were used: a community sample and a non-community sample. The community sample included a random selection of discrete Indigenous communities and associated outstations from the Dwelling Register for Aboriginal and Torres Strait Islander Communities [31]. The survey collected self-reported information about Indigenous Australians’ usual and preferred sources of healthcare [29]. For this study, we focused on services such as Aboriginal Medical Services (AMS), hospitals, general practitioners (GPs), and traditional healers. We initially included data from 4,864 Indigenous Australians aged 18 and over. To make a clear comparison between AMS/Community Clinics (CC) and mainstream GP/hospital services, we excluded people who had no regular healthcare source (*n* = 408) or who used (*n* = 40) and preferred (*n* = 348) a traditional healer. After these exclusions, 4,416 participants were included in the analysis of usual sources of healthcare and 4,712 were included in the analysis of preferred sources of healthcare.

### Outcomes

The study focused on two main questions: where Indigenous Australians usually go when they have a health problem or need advice, and where they would prefer to go if they were sick or needed advice. To analyse this, healthcare options were grouped into two categories: Aboriginal Medical Services (AMS) or Community Clinics (CC), and mainstream general practitioners (GPs) or hospitals. For simplicity, AMS/CC was coded as “1” and mainstream GP/hospital as “2”.

### Explanatory variables

Explanatory variables were broadly categorised into individual- and community-level factors. The individual-level factors included sex, age group, educational status, marital status, employment status, language spoken at home, financial stress, presence of health condition, self-assessed health, satisfaction with own knowledge of culture, removed from the natural family, experienced unfair treatment in the past 12 months, and experienced physical harm in the last 12 months. Community-level factors included are residence (remoteness), the index of relative socioeconomic disadvantage, and the state or territory. Table 1 presents the definitions and classifications of explanatory variables.

**Table 1:** Definitions of variables included in the study

Variables	Definition
**Outcome Variables**	
Usual sources of healthcare	A place where individuals usually go for health problems and advice [56], grouped as “1” = “Aboriginal Medical Service (AMS)/Community clinic (CC)”, and “2” = “mainstream GP/Hospital”.
Preferred sources of healthcare	A place where individuals would like to go if they are sick or need advice about their health [56], and it is grouped as “1” = “Aboriginal Medical Service (AMS)/Community clinic (CC)” and “2” = “mainstream GP/Hospital”
**Explanatory variables**	
***Individual-level factors***	
Sex	Sex was categorised as “1” = “Male”, and “2” = “Female”
Age group	The age of the participants in years was categorised into three categories as “1” = “18–29 years”, “2” = “30–44 years”, and “3” = “45 + years”
Educational status	Educational status was categorised as “1” = “did not complete year 12”, “2” = “completed year 12”, “3” = “trade certificate or diploma”, and “4” = “tertiary education”.
Marital status	Marital status was coded as “1” = “not married”, and “2” = “married”.
Employment status	Employment status was categorised as “1” = “employed”, “2” = “unemployed”, and “3” = “not in the labour force”.
Language spoken at home	The main language spoken at home is grouped as “1” = “English” and “2” = “Other”
Financial stress	Whether a household member could not raise $ 2000 in an emergency: categorised as “1” = “Yes” and “2” = “No”
Presence health condition	A term used to describe the presence of any health condition that is acute or chronic and is categorised as “1” = “Yes” and “2” = “No”
Self-assessed health	Self-assessed health status is a commonly used measure of overall health that reflects a person’s perception of his or her health at a given point in time. Self-rated health was coded as “1” = “Good”, which includes excellent, very good and good or “2” = “Poor”, which includes fair and poor.
Satisfaction with own knowledge of culture	Satisfaction with the level of Indigenous knowledge one possesses and grouped as “1” = “Yes” and “2” = “No”.
Removed from the natural family	It is defined as a participant who has ever been removed from the natural family by the government. Grouped as “1” = “Yes” and “2” = “No”.
Experienced unfair treatment in the past 12 months	Defined as having experiences of discrimination (e.g., called names, racial comments, ignored, not trusted, unfairly arrested, humiliated, having objects thrown at them) because of Indigenous backgrounds in the last 12 months. Grouped as “1” = “Yes” and “2” = “No”.
Experienced physical harm in the last 12 months	Defined as having experiences of being physically hurt or harmed by someone on purpose, including physical fights in the last 12 months. Grouped as “1” = “Yes” or “2” = “No”.
***Community-level factors***	
Residence (Remoteness)	Remoteness measures are calculated using Accessibility/Remoteness Index of Australia (ARIA+) scores, which are based on the road distance from a populated locality to the nearest urban centre [57]. In this study, remoteness is grouped as “1” = “Major city”, “2” = “Inner/outer regional”, and “3” = “Remote or very remote”.
The index of relative socioeconomic disadvantage	The Index of Relative Socio-economic Disadvantage (IRSD) is a general socio-economic index that summarises a range of information about the economic and social conditions of people and households within an area. IRSD is grouped into five groups (quintiles) – from the most disadvantaged (worst off or lowest socioeconomic area) to the least disadvantaged (best off or highest socioeconomic area).
State/Territory	A States or Territory are defined as the largest spatial unit within the Australian Statistical Geography Standard (ASGS) Main Structure [58]. States and Territories are recognised as “1” = “New South Wales”, “2= “Victoria”, 3= “Queensland”, 4= “South Australia”, 5= “Western Australia”, 6= “Tasmania”, 7= “Northern Territory”, and 8= “Australian Capital Territory”

AMS, Aboriginal Medical Services

### Statistical analysis

Data were accessed through the ABS Data Lab (https://www.abs.gov.au/statistics/microdata-tablebuilder/datalab) and analysed using STATA software (version 18, Stata Corp, College Station, TX, USA). The initial analysis summarised categorical variables using frequencies, percentages, means, and standard deviations. The prevalence of usual and preferred sources of healthcare was calculated across each explanatory variable. All frequencies and percentages were weighted using the personal weight variable (‘fingerwt’), with the sample size estimated by dividing the personal weight variable by 100.

The NATSIHS employed a complex sampling design, where individuals are grouped within communities, and communities are grouped within larger geographic areas, specifically Statistical Area level 2 in the present case [32]. This means people in the same area are more likely to share similar characteristics. To account for this, we used a two-level binary logistic regression model that examined both individual and community factors. We tested four models. Model 0 was the empty model with no predictors, used only to measure differences between communities. Model I included only individual factors, while Model II included only community factors. Finally, Model III combined both individual and community factors and was built step by step to identify the best-fitting model. This final model is the one presented in the results.

We reported results as adjusted odds ratios (aOR) with 95% confidence intervals (CIs), considering p-values below 0.05 as significant. We also measured how much variation came from community-level differences using the intra-class correlation coefficient (ICC). A high ICC indicates that where people live plays an important role in healthcare choices [33]. Model comparisons were made using log-likelihood ratio tests, Akaike Information Criterion tests (AIC), and Bayesian information criteria (BIC) values, and Model III was identified as the best fit [34, 35]. Finally, we checked for multicollinearity with the Variance Inflation Factor (VIF) and found no issues [36].

## Results

### Study participants

**Fig. 1:**
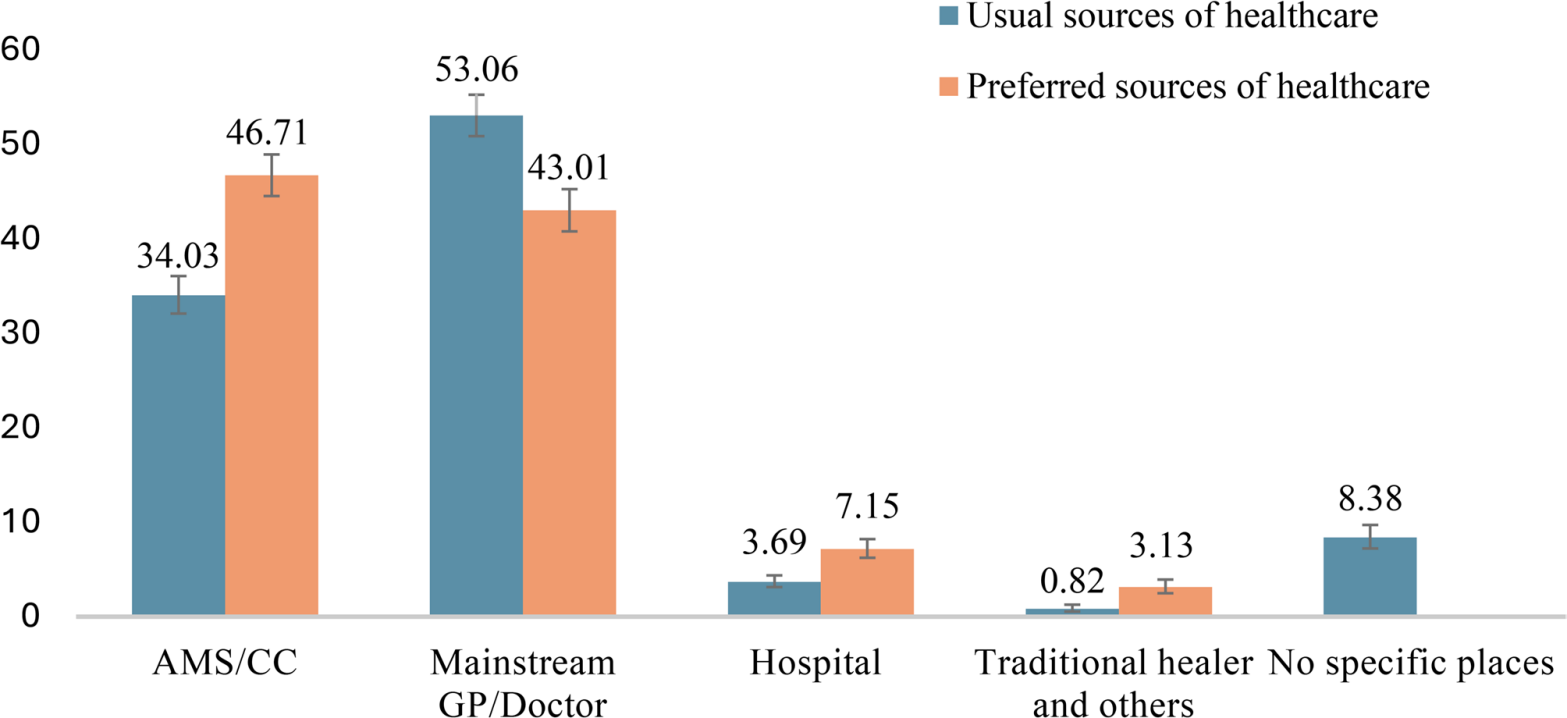
Usual and preferred sources of healthcare for Indigenous Australians

Of the 4,864 Indigenous Australians, more than half (53.1%, *n* = 2,581; 95% CI: 50.9–55.2) usually visited a mainstream GP or doctor, while one-third (34.0%, *n* = 1,656; 95% CI: 32.1–36.1) used an AMS/CC. By contrast, when asked about preference, more participants favoured AMS/CC (46.7%, *n* = 2,272; 95% CI: 44.5–48.9) than mainstream GPs (43.0%, *n* = 2,092; 95% CI: 40.8–45.3). Hospitals accounted for 3.7% (*n* = 179; 95% CI: 3.1–4.4) of usual care and 7.2% (*n* = 348; 95% CI: 6.2–8.2) of preferred care. Traditional healers were used regularly by 0.8% (*n* = 40; 95% CI: 0.5–1.3) and preferred by 3.1% (*n* = 152; 95% CI: 2.5–3.9). In addition, 8.4% (*n* = 408; 95% CI: 7.2–9.7) reported no usual source of healthcare (Fig. 1).

For this study, we analysed Indigenous Australians who identified either an Aboriginal Medical Service/community clinic (AMS/CC) or a mainstream GP/hospital as their usual (*n* = 4,416) or preferred (*n* = 4,712) sources of healthcare. Among those reporting their usual source of care, most (89.7%, *n* = 3,960) spoke English, 60.1% (*n* = 2,656) were unmarried, and 43.8% (*n* = 1,866) had not completed Year 12 education. For participants who indicated their preferred source of care, one-third (33.9%, *n* = 1,597) were from New South Wales, approximately one-quarter (26.9%, *n* = 1,265) from Queensland, and 3.8% (*n* = 179) from Tasmania. In terms of employment, nearly half (48.0%, *n* = 2,264) were employed, while 41.6% (*n* = 1,960) were not in the labour force (Table 2).

**Table 2:** Characteristics of Aboriginal and Torres strait islanders aged ≥ 18 years included in this study

Characteristics	Usual sources of healthcare			Preferred sources of healthcare		
	Male (*N* = 2022)	Female (*N* = 2394)	Total (*N* = 4416)	Male (*N* = 2280)	Female (*N* = 2432)	Total (*N* = 4712)
*Individual-level factors*	n (%)	n (%)	n (%)	n (%)	n (%)	n (%)
Age group (years)						
18–29	705 (34.86)	792 (33.06)	1497(33.89)	845(3704)	825(33.93)	1670(35.43)
30–45	557 (27.53)	672(28.09)	1229(27.83)	630(2764)	693(28.49)	1323(28.08)
45+	760 (37.61)	930(38.85)	1690(38.28)	805(3532)	914(37.58)	1719(36.49)
Highest level of education						
Did not complete year 12	905(46.08)	961(41.80)	1866(43.77)	1004(45.35)	994(42.46)	1998(43.86)
Completed year 12	265(13.48)	325(14.15)	590(13.84)	305(13.81)	336(14.34)	641(14.08)
Trade certificate or diploma	665(33.84)	814(35.40)	1479(34.69)	763(34.46)	805(34.42)	1568(34.44)
Tertiary education	129(6.60)	199(8.65)	328(7.70)	142(6.38)	205(8.78)	347(7.62)
Marital status						
Married	810(40.08)	950(39.67)	1760(39.86)	908(39.83)	945(38.84)	1853(39.32)
Not married	1212(59.92)	1444(60.33)	2656(60.14)	1372(60.17)	1487(61.16)	2859(60.68)
Employment status						
Employed	1058(52.31)	1020(42.64)	2078(47.06)	1232(54.04)	1032(42.41)	2264(48.04)
Unemployed	248(12.26)	196(8.17)	444(10.04)	281(12.33)	207(8.53)	488(10.37)
Not in the labor force	716(35.43)	1178(49.19)	1894(42.89)	767(33.62)	1193(49.06)	1960(41.59)
Language						
English	1808(89.39)	2152(89.92)	3960(89.68)	2060(90.32)	2184(89.81)	4244(90.06)
Other	214(10.61)	242(10.08)	456(10.32)	220(9.68)	248(10.19)	468(9.94)
Financial stress						
Yes	946(49.20)	1258(55.82)	2204(52.77)	1061(48.8)	1285(56.03)	2346(52.51)
No	977(50.80)	995(44.18)	1972(47.23)	1113(51.2)	1008(43.97)	2121(47.49)
Presence of any health condition						
Yes	1714(84.75)	2162(90.33)	3876(87.77)	1906(83.60)	2187(89.93)	4093(86.87)
No	308(15.25)	232(9.67)	540(12.23)	374(16.40)	245(10.07)	619(13.13)
Self-rated health						
Good	1535(75.95)	1755(73.30)	3290(74.51)	1754(76.93)	1786(73.44)	3540(75.13)
Poor	487(24.05)	639(26.70)	1126(25.49)	526(23.07)	646(26.56)	1172(24.87)
Satisfaction with own knowledge of culture						
Unsatisfied	410(20.68)	644(27.08)	1054(24.18)	488(21.80)	655(27.11)	1143(24.55)
Neutral	388(19.58)	468(19.70)	856(19.64)	468(20.90)	474(19.63)	942(20.25)
Satisfied	1185(59.74)	1265(53.22)	2450(56.18)	1284(57.30)	1286(53.26)	2570(55.20)
Removed from the natural family						
Yes	267(14.17)	393(17.17)	660(15.81)	293(13.78)	391(16.80)	684(1536)
No	1621(85.83)	1897(82.83)	3518(84.19)	1837(86.22)	1935(83.20)	3772(84.64)
Experienced unfair treatment in the past 12 months						
Yes	450(24.46)	564(25.24)	1014(24.89)	494(23.65)	570(25.04)	1064(24.38)
No	1390(75.54)	1669(74.76)	3059(75.11)	1593(76.35)	1704(74.96)	3297(75.62)
Experienced physical harm in the last 12 months						
Yes	120(6.08)	140(6.00)	260(6.04)	135(6.10)	145(6.13)	280(6.12)
No	1842(93.92)	2194(94.00)	4036(9396)	2076(93.90)	2223(93.87)	4299(93.88)
**Community-level variable**						
Residence (Remoteness)						
Major city	751(37.16)	898(37.50)	1649(3734)	868(38.05)	900(37.01)	1768(37.51)
Inner and outer regional	857(42.36)	1030(43.02)	1887(4272)	978(42.92)	1057(43.46)	2035(43.20)
Remote and very remote	414(20.48)	466(19.48)	880(1994)	434(19.03)	475(19.53)	909(19.29)
The index of relative socioeconomic disadvantage						
Quintile 1 (most disadvantaged)	1065(52.65)	1313(54.82)	2378(53.83)	1178(51.64)	1353(55.64)	2531(53.70)
Quintile 2	409(20.20)	450(18.81)	859(19.44)	483(21.16)	452(18.60)	935(19.84)
Quintile 3	276(13.66)	338(14.13)	614(13.92)	314(13.78)	332(13.65)	646(13.71)
Quintile 4	138(06.84)	217(09.08)	355(08.05)	162(7.13)	218(8.95)	380(8.07)
Quintile 5 (least disadvantaged)	134(06.65)	76(03.16)	210(04.76)	143(6.29)	77(3.16)	220(4.68)
State/Territory						
New South Wales	670(33.14)	814(33.99)	1484(33.60)	767(33.63)	830(34.11)	1597(33.88)
Victoria	147(7.27)	165(6.88)	312(7.06)	170(7.46)	175(7.20)	345(7.33)
Queensland	552(27.27)	655(27.37)	1207(27.33)	617(27.09)	648(26.63)	1265(26.85)
South Australia	94(4.66)	125(5.23)	219(4.97)	119(5.22)	127(5.22)	246(5.22)
Western Australia	255(12.61)	292(12.20)	547(12.39)	280(12.26)	299(12.31)	579(12.29)
Tasmania	77(3.78)	90(3.76)	167(3.77)	87(3.81)	92(3.79)	179(3.80)
Northern Territory	209(10.33)	229(9.58)	438(9.93)	217(9.50)	238(9.79)	455(9.64)
Australian Capital Territory	18(0.93)	24(0.98)	42(0.96)	23(1.03)	23(0.95)	46(0.99)

Note that the difference between the total sample size and certain variables’ frequencies is due to missing values

### Determinants of usual sources of healthcare

Our findings showed that Indigenous Australians who were not partnered were more likely to use AMS/CC than those who were partnered (aOR = 1.67, 95% CI: 1.27–2.19). Significantly higher use was also observed among those experiencing financial stress (aOR = 2.25, 95% CI: 1.71–2.96), those satisfied with their cultural knowledge (aOR = 1.90, 95% CI: 1.38–2.61), and those who had been removed from their natural family (aOR = 1.83, 95% CI: 1.33–2.52). Experiencing unfair treatment in the past 12 months increased the odds of using AMS/CC by 60% (aOR = 1.60, 95% CI: 1.20–2.13).

Location strongly influenced use. Indigenous Australians living in Queensland (aOR = 2.62, 95% CI: 1.40–4.92), the Northern Territory (aOR = 6.78, 95% CI: 3.39–13.53), and the ACT (aOR = 2.56, 95% CI: 1.18–5.55) were more likely to use AMS/CC compared to those in New South Wales, while those in Tasmania were 75% less likely (aOR = 0.25, 95% CI: 0.08–0.72). Living in inner or outer regional areas (aOR = 3.26, 95% CI: 2.20–4.82) or very remote areas (aOR = 8.01, 95% CI: 4.70–13.63) greatly increased use. Finally, Indigenous Australians living in less disadvantaged areas (quintile 4) were 65% less likely to use AMS/CC than those in the most disadvantaged areas (quintile 1, aOR = 0.35, 95% CI: 0.16–0.74) (Table 3).

**Table 3:** Individual- and community-level factors associated with AMS/CC being the usual and/or preferred sources of healthcare in indigenous Australians aged 18 years and above

Variables	Usual sources of healthcare		Preferred sources of healthcare	
	aOR [95% CI]	*P*-value	aOR [95% CI]	*P*-value
**Individual-level variable**				
Sex				
Male	ref		ref	
Female	1.05[0.82, 1.33]	0.686	1.54[1.28, 1.84] *	0.000
Age group (years)				
18–29	ref		ref	
30–45	1.08[0.79, 1.49]	0.600	0.93[0.74, 1.17]	0.569
45+	0.90[0.65, 1.26]	0.573	0.71[0.56, 0.91] *	0.007
Highest level of education				
Did not complete year 12	ref		ref	
Completed year 12	0.95[0.64, 1.39]	0.801	1.03[0.78, 1.38]	0.788
Trade certificate or diploma	1.05[0.78, 1.41]	0.714	1.24[0.99, 1.54]	0.052
Tertiary education	0.91[0.53, 1.54]	0.730	1.15[0.78, 1.67]	0.465
Marital status				
Married	ref		ref	
Not married	1.67[1.27, 2.19] *	0.000	1.26 [1.04, 1.54] *	0.017
Employment status				
Unemployed	ref		ref	
Employed	0.70[0.46, 1.08]	0.114	0.80[0.59, 1.10]	0.189
Not in the labour force	1.09[0.72, 1.65]	0.667	1.14[0.83, 1.56]	0.397
Language				
English	ref		ref	
Other	1.82[0.99, 3.32]	0.051	1.22[0.76, 1.94]	0.404
Financial stress				
Yes	2.25[1.71, 2.96] *	0.000	1.60[1.30, 1.97] *	0.000
No	ref		ref	
Presence of any health condition				
Yes	ref		ref	
No	1.04[0.71, 1.53]	0.808	0.98[0.74, 1.31]	0.928
Self-rated health				
Good	ref		ref	
Poor	0.88[0.66, 1.18]	0.417	1.14[0.91, 1.42]	0.224
Satisfaction with own knowledge of culture				
Unsatisfied	ref		ref	
Neutral	1.09[0.74, 1.61]	0.649	0.73[0.68, 1.05]	0.132
Satisfied	1.90[1.38, 2.61] *	0.000	1.40[1.11, 1.76] *	0.004
Removed from the natural family				
Yes	1.83[1.33, 2.52] *	0.000	1.70[1.32, 2.18] *	0.000
No	ref		ref	
Experienced unfair treatment in the past 12 months				
Yes	1.60[1.20, 2.13] *	0.001	1.68[1.35, 2.08] *	0.000
No	ref		ref	
Experienced physical harm in the last 12 months				
Yes	1.07[0.67, 1.72]	0.763	0.77[0.53, 1.11]	0.167
No	ref		ref	
**Community-level variable**				
Residence (Remoteness)				
Major city	ref		ref	
Inner and outer regional	3.26[2.20, 4.82] *	0.000	1.43[1.01, 2.03] *	0.039
Remote and very remote	8.01[4.70, 13.63] *	0.000	2.86[1.66, 4.92] *	0.000
The index of relative socioeconomic disadvantage				
Quintile 1 (most disadvantaged	ref		ref	
Quintile 2	0.78[0.49, 1.22]	0.283	0.96[0.70, 1.33]	0.840
Quintile 3	0.60[0.34, 1.06]	0.080	1.11[0.75, 1.64]	0.585
Quintile 4	0.35[0.16, 0.74] *	0.006	1.01[0.63, 1.63]	0.940
Quintile 5 (least disadvantaged)	0.70[0.25, 1.96]	0.502	0.91[0.46, 1.80]	0.806
State/Territory				
New South Wales (NSW)	ref		ref	
Victoria	1.10[0.51, 2.34]	0.800	0.94[0.58, 1.55]	0.836
Queensland	2.62[1.40, 4.92] *	0.003	1.48[0.97, 2.26]	0.068
South Australia (SA)	1.40[0.59, 3.33]	0.444	0.87[0.48, 1.59]	0.672
Western Australia (WA)	1.37[0.63, 2.99]	0.419	0.89[0.52, 1.49]	0.663
Tasmania	0.25[0.08, 0.72] *	0.010	0.42[0.21, 0.85] *	0.017
Northern Territory (NT)	6.78[3.39, 13.53] *	0.000	2.37[1.17, 4.78] *	0.016
Australian Capital Territory (ACT)	2.56[1.18, 5.55] *	0.017	0.96[0.35, 2.64]	0.949

aOR, Adjusted Odds Ratio: CI, Confidence Interval

### Determinants of preferred sources of healthcare

Indigenous Australians who were female (aOR = 1.54, 95% CI: 1.28–1.84) or unmarried (aOR = 1.26, 95% CI: 1.04–1.54) were more likely to prefer AMS/CC. In contrast, adults aged 45 and older were 29% less likely to prefer AMS/CC compared to those aged 18–29 (aOR = 0.71, 95% CI: 0.56–0.91). Preference for AMS/CC was also higher among those satisfied with their cultural knowledge (aOR = 1.40, 95% CI: 1.11–1.76), those removed from their natural family (aOR = 1.70, 95% CI: 1.32–2.18), and those who had experienced unfair treatment (aOR = 1.68, 95% CI: 1.35–2.08). Location was important as well. Indigenous Australians living in the Northern Territory had higher odds of preferring AMS/CC (aOR = 2.37, 95% CI: 1.17–4.78), while those in Tasmania had lower odds (aOR = 0.42, 95% CI: 0.21–0.85) compared with New South Wales. Similarly, preference was greater among those living in inner or outer regional areas (aOR = 1.43, 95% CI: 1.01–2.03) and very remote areas (aOR = 2.86, 95% CI: 1.66–4.92) (Table 3).

### Random effects (measures of variation) analysis results

The result of the random effects model indicates that there was statistically significant variation in the use and preference of AMS/CC across the clusters. According to the Intra-class correlation (ICC) values, 68.6% of the total variance for usual healthcare and 41.1% of the total variance for preference were attributed to the context of the communities (clusters) where Indigenous people resided. Moreover, the model fit statistics showed a decrease in the values of AIC and BIC across models, indicating progressive improvements in model fitness. Therefore, the final (combined) model that incorporated individual and community-level factors was deemed most suitable for predicting AMS/CC as the usual and preferred healthcare sources among Indigenous communities (Table 4).

**Table 4:** Random effects (measures of variation)

	Model 0	Model I	Model II	Model III
**Usual healthcare setting**				
***Random effect***				
Clusters Variance (95% CI)	7.21[5.75, 9.03]	5.74[4.45, 7.41]	4.00[3.15, 5.08]	3.91[3.00, 5.08]
ICC (%)	68.67	63.58	54.91	54.31
PCV (%)	Reference	20.38	44.52	45.77
***Model fitness***				
Log-likelihood	-1903.3702	-1410.1375	-1793.6093	-1334.7373
AIC	3810.74	2860.275	3617.219	2735.475
BIC	3824.117	2989.559	3717.545	2948.794
**Preferred healthcare setting**				
***Random effect***				
Clusters Variance (95% CI)	2.29[1.86, 2.83]	2.09[1.64, 2.65]	1.78[1.42, 2.23]	1.85[1.45, 2.37]
ICC (%)	41.13	38.88	35.19	36.08
PCV (%)	Reference	8.73	22.27	19.21
***Model fitness***				
Log-likelihood	-2793.3467	-2157.4683	-2749.1012	-2133.5291
AIC	5590.693	4354.937	5528.202	4333.058
BIC	5604.17	4585.260	5629.274	4548.092

ICC = Intra-Class Correlation; PCV=proportional change in variance; CI = Confidence Interval; AIC = Akaike’s Information Criterion; BIC = Schwarz’s Bayesian Information Criteria; **Model 0** is the null model, a baseline model without any determinant variable; **Model I** is adjusted for individual-level factors; **Model II** is adjusted for community-level factors; **Model III** is the final model adjusted for individual- and community-level factors

## Discussion

The study found that while many Indigenous Australians usually access mainstream GPs, they often prefer AMSs, highlighting a mismatch between actual use and preferred source of healthcare. Higher use of and preference for AMS/CC were observed among individuals experiencing financial stress, those with access to cultural knowledge, those who had been removed from their natural family, those facing discrimination, and residents of regional, remote, or Northern Territory areas. In contrast, lower use of and preference for AMSs/CC were observed in less disadvantaged areas and in Tasmania.

Although there is a strong preference for AMS/CCs due to their holistic and culturally informed approach that accounts for physical health as well as social, emotional, and spiritual well-being [37], their actual utilisation remains relatively low. This underutilisation may be associated with a range of structural barriers, including limited access to AMS, shortages of Indigenous staff, fragmented and inconsistent funding arrangements across federal, state, and local governments, and difficulties reaching people living in poverty or with complex social disadvantages who may face accessing mainstream services [38]. Evidence indicates that the number of Indigenous primary healthcare organisations declined from 211 to 198 between 2008 and 09 and 2017–18, and that one in five Indigenous Australians who preferred AMS care reported no local access [9, 39]. Moreover, in 29 areas with at least 200 Indigenous residents, Indigenous-specific primary healthcare services were located more than an hour’s drive away [40]. Workforce challenges have also been reported, with Indigenous staff comprising 51% of employees in Commonwealth-funded Indigenous primary healthcare organisations in 2022 [41, 42].

These gaps highlight the potential importance of further government investment in AMS. For instance, although mainstream services are already funded, evidence suggests they may not adequately reach people experiencing poverty or geographic isolation [43]. Expanding AMS capacity may improve access to culturally safe and holistic care for those who might otherwise face barriers to accessing services, particularly in rural and remote communities. Accordingly, continued efforts may be needed to address these challenges, such as expanding access to high-quality AMS and implementing digital health initiatives supported by sustained funding to strengthen the Indigenous health workforce across states.

This study revealed that access to cultural knowledge and adverse past experiences, such as experiencing discrimination and being removed from one’s natural family, were consistently associated with both the use of and preference for AMS/CC. Evidence suggests that Individuals who have experienced historical and institutional racism may feel more respected and understood in AMS settings, where staff are trained in culturally appropriate practices and may also be members of the community [44]. Moreover, strengthening the capacity of AMSs to collect and utilise health service data could support the review of culturally safe healthcare delivery, performance assessment, and monitoring of patients’ health status [23].

Beyond AMS, customising mainstream health systems to be culturally safe and responsive to the needs of Indigenous people is also important for efforts to close the gap [45, 46]. Mainstream primary healthcare providers may enhance recognition of their Indigenous patients through clinical quality-improvement initiatives and by partnering with the community-controlled sector to monitor performance and value across both sectors [47]. This may include engaging Indigenous communities in the decision-making process and incorporating cultural safety practices to ensure more equitable and respectful care [47].

While cultural knowledge and adverse experiences were consistently associated with both the use of and preference for AMS/CC, certain demographic factors (sex and age) appeared to have a stronger association with preference than with actual use. This higher preference among females may be due to the gender-sensitive care and sense of comfort that AMSs can offer when addressing sensitive issues such as reproductive health, parenting, and family violence [48]. However, this higher preference is not reflected in actual utilisation, which may indicate barriers to accessing AMS/CC services. Conversely, the lower preference for AMS among older individuals may be related to the limited availability of advanced or specialist medical care at these centres. Older individuals may perceive that mainstream health facilities provide more comprehensive or immediate care for complex health conditions than AMS, as mainstream healthcare settings are often better equipped with diagnostic tools, emergency services, and specialist staff that may not be available in AMSs, particularly in remote or underserved areas [49]. For instance, in 2017–18, Indigenous primary health care organisations reported service gaps in areas such as mental health and social and emotional health and wellbeing, as well as alcohol, tobacco, and other drug services [9]. These findings highlight the importance of a deeper understanding of the diverse needs and preferences of Indigenous communities, alongside continued investment and support to strengthen the delivery of AMS services.

Our findings indicate that Indigenous Australians living in rural and regional areas were more likely to prefer and use AMSs. This is supported by prior studies showing that geographic isolation has been associated with delayed care, higher costs, and reduced continuity of healthcare [50, 51]. Moreover, AMSs may be well-suited to serve these communities as they aim to provide culturally safe and holistic care that addresses physical, social, emotional, and cultural well-being, and are often staffed by Indigenous professionals who can help foster trust and understanding with patients [52]. Therefore, expanding AMS coverage in underserved rural and remote areas helps to improve access to care and support more equitable health service delivery for Indigenous Australians.

In this study, preferences for and use of AMS/CCs among Indigenous people varied substantially across states and territories, likely reflecting differences in the availability and accessibility of AMS/CC services. Evidence indicates that the Northern Territory and Queensland have a relatively higher density of AMSs and larger Indigenous populations, which may have enhanced access to AMS care, whereas Tasmania has fewer AMSs and a smaller Indigenous population, which may have limited both the use of and preference for AMSs services [53]. Additionally, individuals living in the fourth socioeconomic quintile (relatively less disadvantaged) were less likely to use AMS/CCs, suggesting that AMS/CCs services are more frequently accessed by those residing in more socioeconomically disadvantaged areas. Variation by state and index of relative socioeconomic status may reflect differences in health care infrastructure as well as the extent of coordination and policy implementation across jurisdictions [54, 55]. These findings underscore the importance of continued government investment in AMSs to improve access for rural, remote, and socioeconomically disadvantaged populations who may have limited access to mainstream health services.

This study has several strengths. To our knowledge, it is the first to investigate the individual and community-level factors influencing the choice of healthcare sources among Indigenous Australians. We employed a rigorous multilevel modelling approach, which allowed us to account for variation across clusters/communities and ensured that our findings are reliable and can effectively inform national and local policy decisions in Australia. However, our results should also be interpreted with caution. First, the use of cross-sectional data limits our ability to draw causal inferences between the independent variables and healthcare choices; the results, therefore, reflect associations rather than cause-and-effect relationships. Second, the study relies on self-reported information, such as their experience of unfair treatment and physical harm, which may introduce recall and social desirability bias. Third, since the study used data from the 2018–19 NATSIHS, patterns in the use of and preferences for healthcare sources may have changed since then. Fourth, some respondents may not clearly distinguish between different categories of healthcare settings, and therefore, it is unclear to what extent respondents were referring specifically to mainstream health services (general practitioners and hospitals) or to Indigenous-specific primary healthcare services (AMS).

In conclusion, most Indigenous Australians continue to access mainstream GPs’ services rather than AMS’s services. This probably reflects the limited availability and accessibility of AMSs in some areas, which leaves people with little option but to seek care from a GP. Most factors associated with the use of AMS/CC also showed similar patterns with preference for AMS/CC, although the strength of these associations varied. The findings highlight the need to expand AMSs, particularly in rural, regional areas and socioeconomically disadvantaged areas, or to customise mainstream healthcare to ensure services are culturally appropriate, respectful, and aligned with the values and preferences of Indigenous communities.

## Data Availability

The Australian Bureau of Statistics (ABS) stores de-identified, unit record-level data that underlie national surveys on its DataLab online platform. Users can perform more detailed analyses, such as generating publicly unavailable variables, and all results are vetted by ABS staff before release. Access to ABS TableBuilder and the ABS DataLab requires formal approval processes ([https://www.abs.gov.au/statistics/microdata-tablebuilder/datalab] (https://www.abs.gov.au/statistics/microdata-tablebuilder/datalab))
